# Three-Dimensional Enlow’s Counterpart Analysis: Neutral Track

**DOI:** 10.3390/diagnostics13142337

**Published:** 2023-07-11

**Authors:** Antonino Peluso, Giulia Falone, Rossana Pipitone, Francesco Moscagiuri, Francesco Caroccia, Michele D’Attilio

**Affiliations:** Department of Innovative Technologies in Medicine & Dentistry, University “G. d’Annunzio” Chieti-Pescara, 66100 Chieti, Italyfrancesco.moscagiuri@unich.it (F.M.);

**Keywords:** anthropometry, cephalometry, cone-beam computed tomography, dental diagnosis, radiography, orthodontics

## Abstract

The aim of this study is to provide a novel method to perform Enlow’s neutral track analysis on cone-beam computed tomography (CBCT) images. Eighteen CBCT images of skeletal Class I (ANB = 2° ± 2°) subjects (12 males and 6 females, aged from 9 to 19 years) with no history of previous orthodontic treatment were selected. For each subject, 2D Enlow’s neutral track analysis was performed on lateral cephalograms extracted from CBCT images and 3D neutral track analysis was performed on CBCT images. A Student’s *t*-test did not show any statistically significant difference between the 2D and 3D measurements and therefore the method proposed by this study to realize the neutral track analysis on 3D images is valid and superimposable on that described by Enlow on lateral cephalograms. Further studies with a large sample and different skeletal class subjects are needed to confirm the results of this research.

## 1. Introduction

Cephalometric analysis is a common procedure performed on lateral cephalograms in order to assess the craniofacial growth pattern and the type of malocclusion in sagittal and vertical directions. Despite its great usefulness, it has several limitations: first of all, the analysis depends on the accurate and repeatable definition of a standardized head position [1], difficulties in determining the difference between the left and right sides for superimposed structures, the approximation of measurements in the presence of facial asymmetries [2] and finally the fact that deformities in the midfacial area cannot be detected [3].

The need to study bone quality in surgical and non-surgical planning has led to the use of cone beam computed tomography (CBCT) in dentistry.

Thanks to its characteristics of high resolution, low radiation exposure and low cost, CBCT has replaced the medical CT that was commonly used. Although the assessment of bone quality has been questioned by recent studies [4], CBCT remains of great utility and wide use in various dental disciplines.

The use of CBCT in common daily practice can help orthodontists to overcome all the common bidimensional cephalometric limitations thanks to the 3D visualization of skeletal structures and high bone contrast [5].

Two systematic reviews by Smektala et al. and [6] Pittayapat et al. [7] concluded that there is still some criticism regarding the accuracy of the methods proposed so far.

Among the previously proposed methods for 3D cephalometry, only one study by D’Attilio at al. [8] described the application of Donald H. Enlow’s concepts on 3D images; however, this study only focused on the description of the cephalometric points included in Enlow’s horizontal counterpart analysis. The complete Enlow’s analysis is composed of horizontal counterpart analysis, vertical counterpart analysis and neutral track analysis, so the complete 3D Enlow’s analysis is still missing.

The neutral track analysis allows us to evaluate the “rotational factor” in the craniofacial development and growth [9,10] of the following structures: the middle cranial floor, mandibular ramus, occlusal plane and nasomaxillary complex. The rotational factor is evaluated by realizing an individual track on the patient lateral cephalogram and comparing it with an ideal track, defined as neutral. The only normal value in the neutral track is the angle between the middle cranial floor (MCF) and the pterygo-maxillary plane (PM) which must be 40.3°; this value is fundamental in the realization of the neutral track.

Consistent with this scientific evidence and following the recent investigation by D’Attilio at al. [8], the aim of this study is to provide a method to perform the analysis of Enlow’s neutral track in 3D. Particularly, a 3D individual track was defined and a method of how to realize the 3D neutral track is provided.

## 2. Materials and Methods

Eighteen CBCT images of skeletal Class I (ANB = 2° ± 2°) subjects (12 males and 6 females, aged from 9 to 19 years) with no history of previous orthodontic treatment were selected from the archives of the Unit of Orthodontics, Department of Innovative Technologies in Medicine and Dentistry, University of Chieti, Chieti, Italy. The radiographic images were obtained by a low-dose CBCT machine (Vatechlpax 3D PCH-6500, Fort Lee, NJ, USA) and processed using the Ez3D Plus Software (Vatech, Global Fort Lee, NJ, USA). The scanning procedure was previously described by Moscagiuri et al. [11]. For each subject, 2D Enlow’s neutral track analysis [10] was performed on lateral cephalograms extracted from CBCT images using the OrisCeph3 (OrisLine; Elite Computer Italia S.r.l., Milano, Italy) software and 3D neutral track analysis was performed on CBCT images using the Materialise Mimics Software (Materialise NV, Leuven, Belgium).

### 2.1. Two-Dimensional Neutral Track Analysis

#### 2.1.1. Two-Dimensional Individual Track

**Figure 1:**
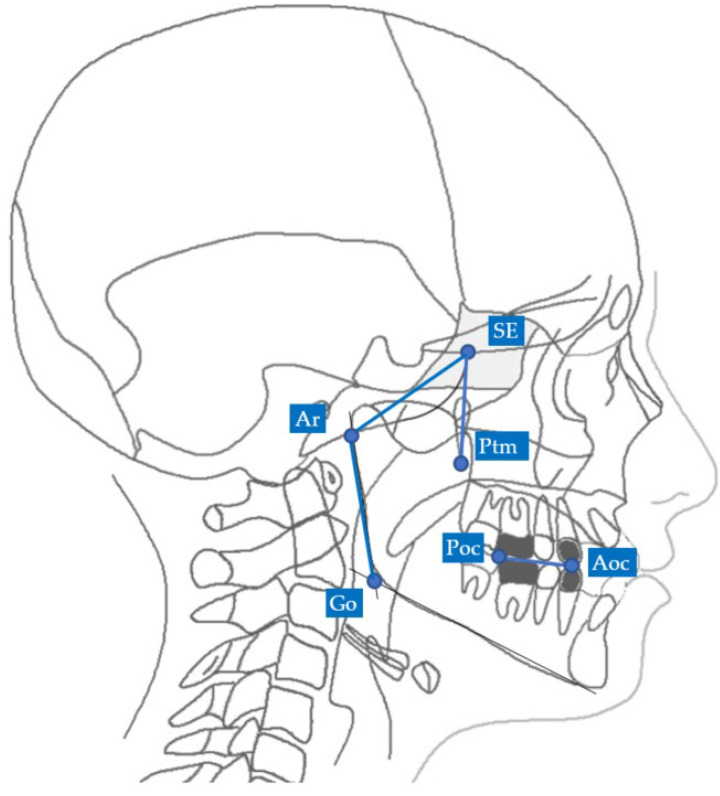
Points and planes of the 2D individual track. For landmark description, see Table 1.

The 2D neutral track analysis compares an individual track with a neutral track [10] The individual track is identified by the following lines: the middle cranial floor, pterygo-mandibular plane, mandibular ramus and functional occlusal plane. All the landmarks and lines of the individual track are explained in Table 1 and all the patient individual planes are shown in Figure 1.

#### 2.1.2. Two-Dimensional Neutral Track

**Figure 2:**
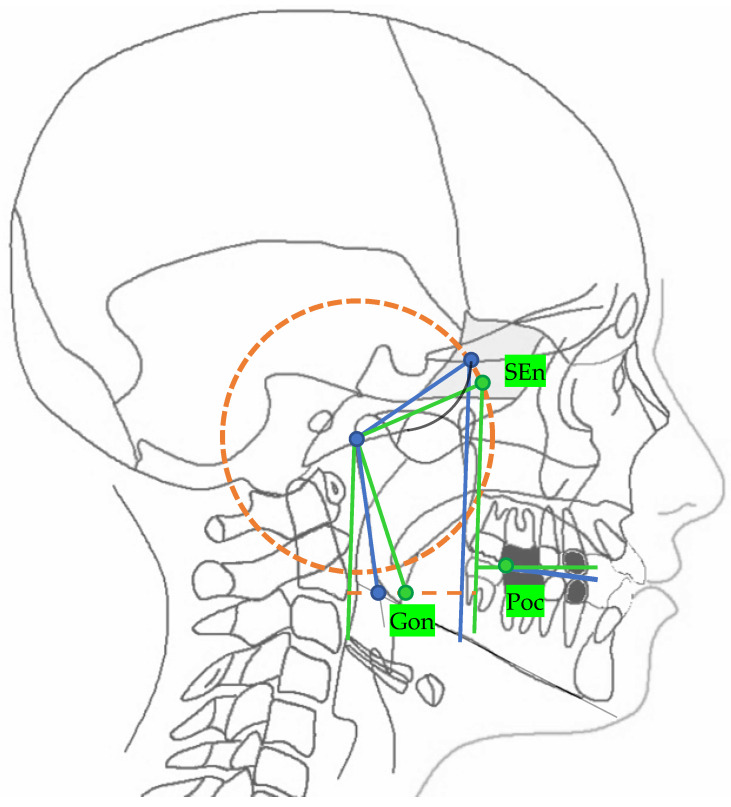
In green: the 2D neutral track. For landmark description, see Table 2.

The neutral track (Figure 2) is drawn on the lateral cephalograms identifying the neutral points and planes explained in Table 2.

The only normal value in the neutral track is the angle between the neutral middle cranial floor plane (MCFn) and neutran pterygo-mandibular plane (PMn) which has a value of 40.3° and from which the realization of the neutral track depends.

### 2.2. Three-Dimensionla Neutral Track Analysis

#### 2.2.1. Three-Dimensional Individual Track

Enlow’s 3D individual track was identified on CBCT images with the points explained in Table 3.

**Figure 3:**
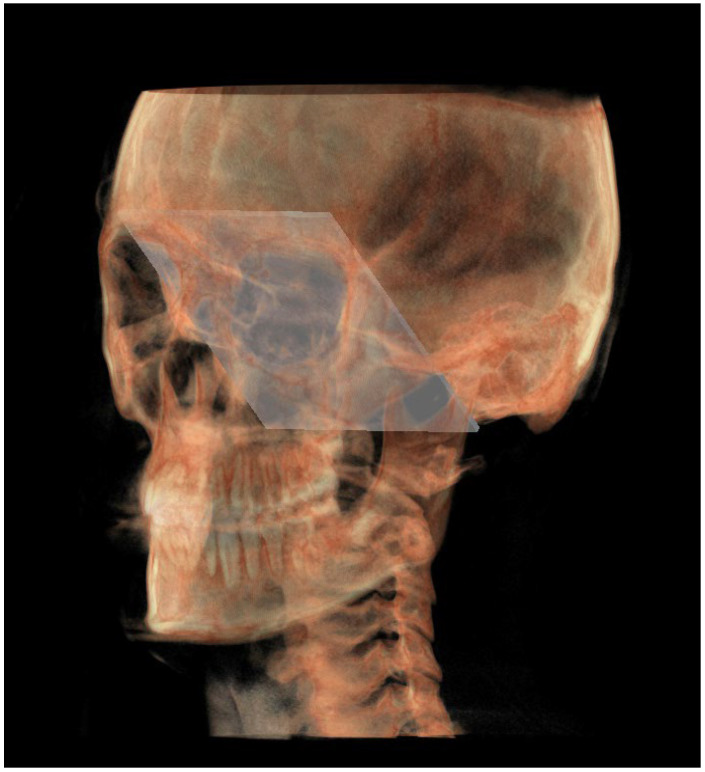
Middle cranial floor.

**Figure 4:**
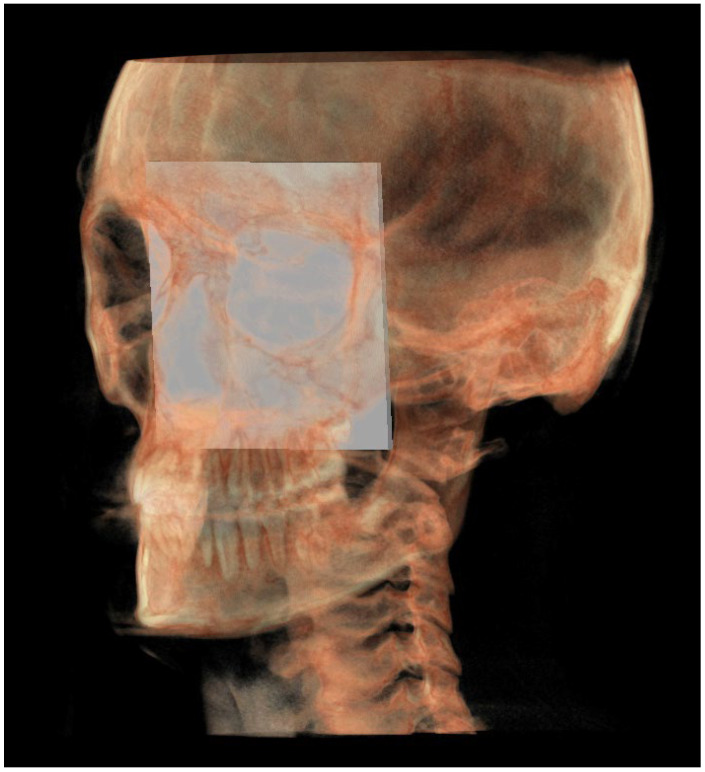
Pterygo-mandibular plane.

**Figure 5:**
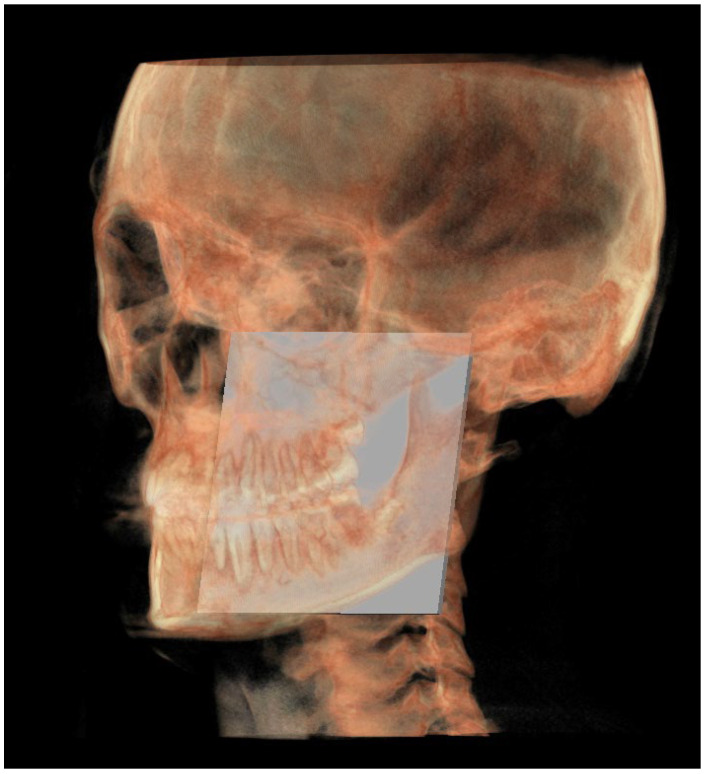
Mandibular ramus plane.

**Figure 6:**
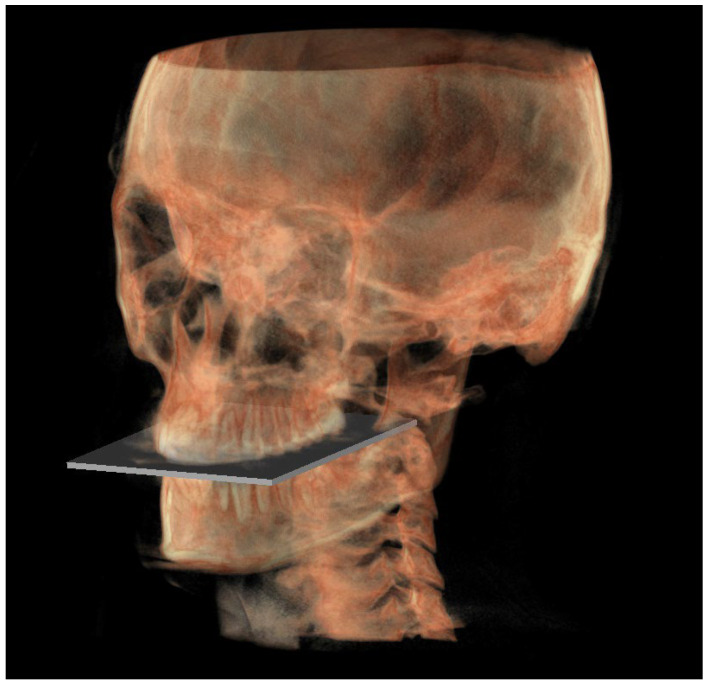
Functional occlusal plane.

The planes of the individual track previously described in 2D were identified in 3D according to the geometric rule of plane construction of a “plane passing through 3 points”:

○ The MCF (Figure 3) was identified by the points middle cranial floor right side (rMCF) and middle cranial floor left side (lMCF) [12] and Basion (Ba);
○ PM (Figure 4) was identified by the points lMCF, rMCF and posterior nasal spine (PNS);
○ The mandibular ramus plane (MR) (Figure 5) was identified by the following points: the middle points between the right and left condylion (mCo) and the right Gonion (rGo) and left Gonion (lGo). To adequately locate the mCo, it is advisable to draw a line on the frontal view from the right condylion to the left one to have a reference along which measure the mid-distance;
○ The functional occlusal plane (FOP) (Figure 6) was identified by the posterior occlusal contact right side (rPoc), posterior occlusal contact left side (lPoc) and the middle point between the right and left anterior occlusal contact (mAoc). To adequately locate the mAoc, it is advisable to draw a line on the transversal view from the right mesial premolar contact to the left one to have a reference along which measure the distance.

**Figure 7:**
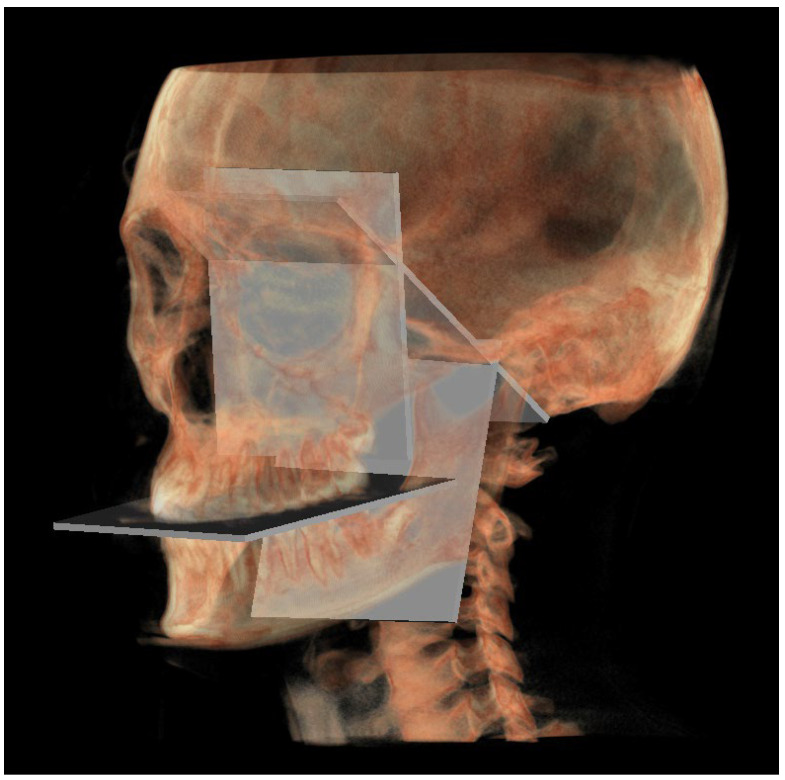
All the planes of the 3D individual track.

A representation of all 3D individual track planes (Figure 7).

#### 2.2.2. Three-Dimensional Neutral Track

The individual track is then compared with a neutral track; the points and planes used in the construction of the neutral track are listed in Table 4 and Table 5 and afterwards a method of how to construct the 3D neutral track will be explained.

**Figure 8:**
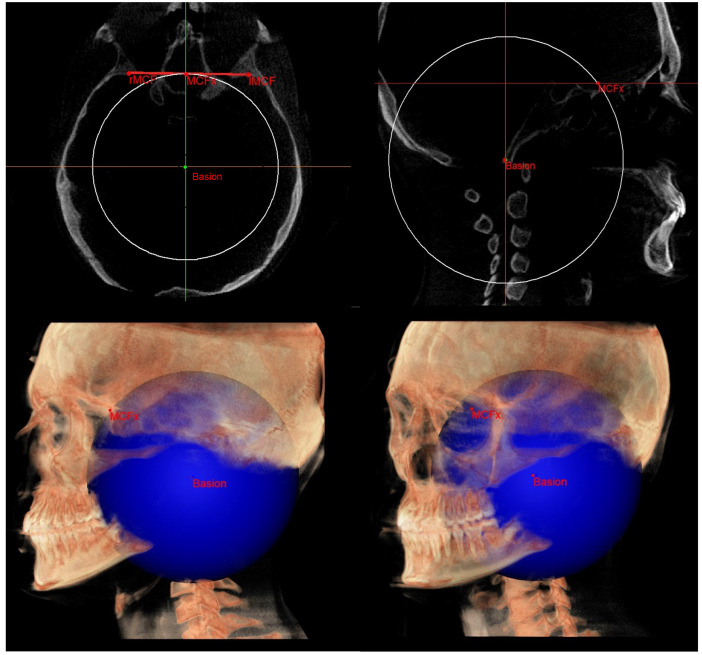
Sphere with radius from Ba to MCFx.

The construction of the neutral track [10] began by drawing a sphere with its center in the Ba point and the radius corresponding to the segment that joins the Ba to the middle point MCF (MCFx) (Figure 8). From this sphere in the sagittal view, we obtained a circumference necessary to identify the neutral sphenoethmoidal junction point (SEn).

**Figure 9:**
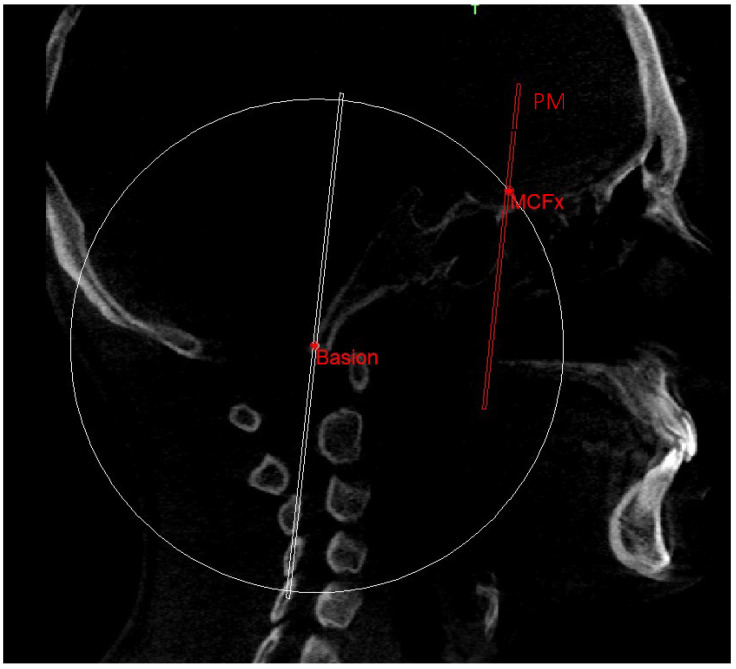
CPBa.

In order to localize the SEn point, the geometric construction rule of “parallel lines cut by a transversal line” was used: according to this rule, if two parallel lines are cut by a transversal one, alternate internal angles equal to each other are formed. Therefore, on the sagittal slice where the Ba point is located, we proceeded to trace a plane passing through Ba and parallel to the PM, defined as the construction plane through Ba (CPBa) (Figure 9).

**Figure 10:**
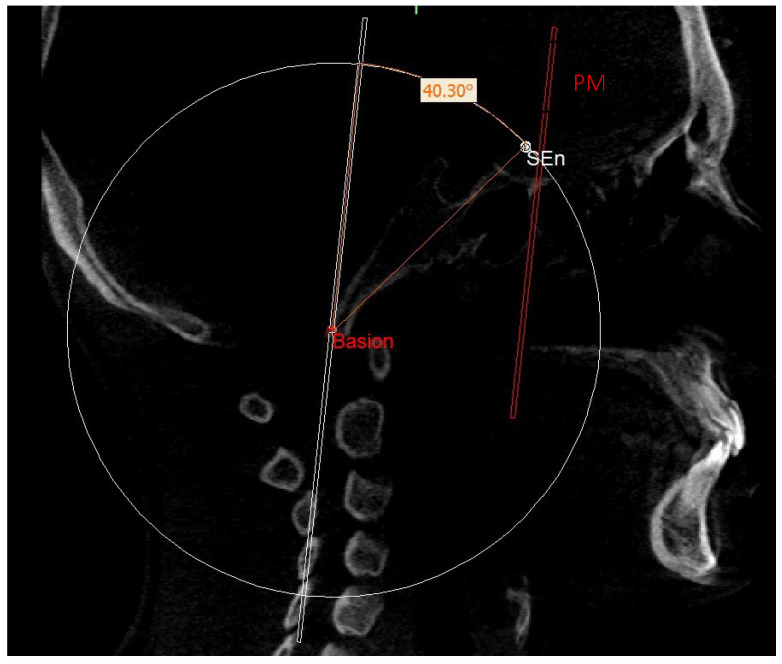
SEn.

This plan was not directly involved in the definition of the neutral 3D track but was used for the construction of the latter. In fact, an angle of 40.3° was built on this plane so that one side lies on the construction plane and the other side intersects with the circumference. The point of intersection with the circumference identified the SEn (Figure 10).

**Figure 11:**
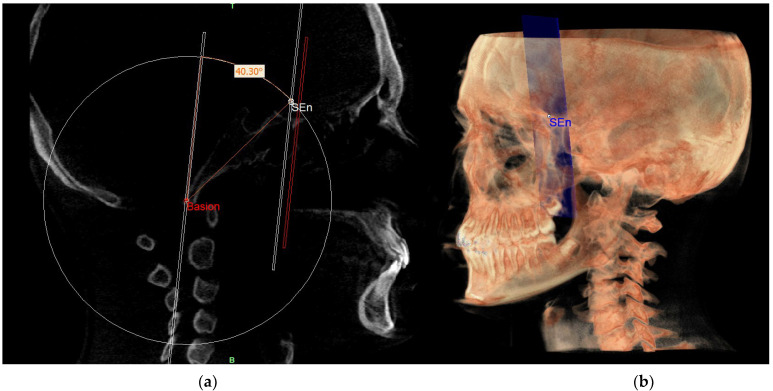
PMn plane and SEn point in the sagittal view (**a**) and on volume rendering (**b**).

This point was used to draw the PMn plane which is parallel to the PM plane. In this way, CPBa and PMn represent two parallel lines cut by the BaSEn line and the internal angle that this latter forms with the PMn is 40.3° (Figure 11).

**Figure 12:**
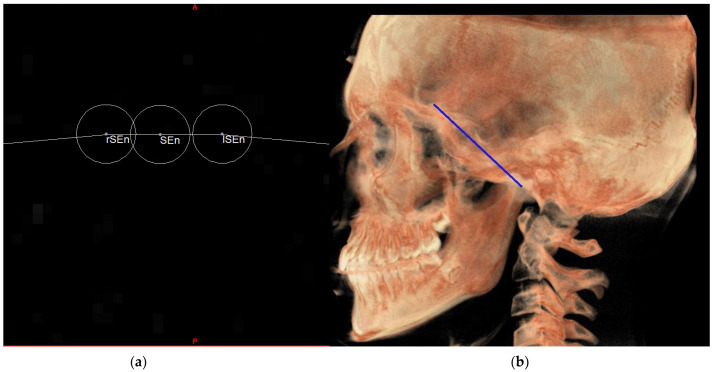
rSEn and lSEn points on the axial view (**a**) and MCFn plane on volume rendering (**b**).

To draw the plane corresponding to the MCFn, three points are needed. Knowing the SEn, in an axial view we proceeded to identify two other points on the circumference arbitrarily positioned on the right and left sides of SEn which are defined as the right SE neutral (rSEn) and left SE neutral (lSEn), respectively. These two points together with the point Ba define the MCF neutral plane (Figure 12).

Subsequently, the points neutral right gonion (rGon) and neutral left gonion (lGon) were identified. These points, together with the mCo, were used to realize the neutral mandibular ramus plane (MRn).

**Figure 13:**
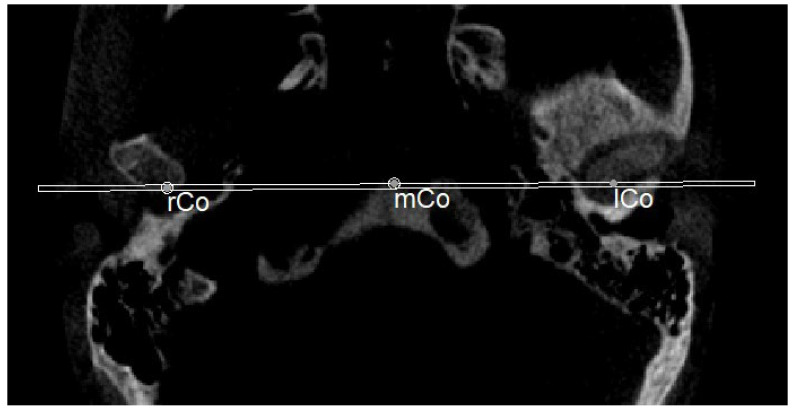
CPCo.

**Figure 14:**
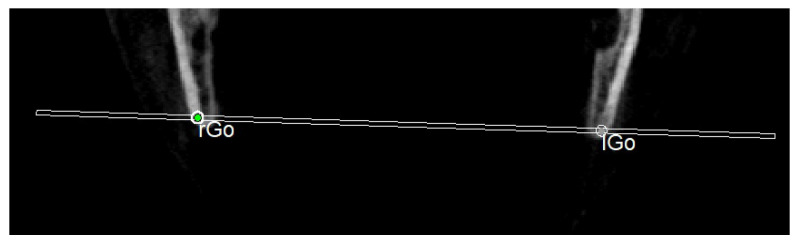
GoP.

To identify the rGon and lGon, it was necessary to construct two reference planes, namely a construction plane through condylion (CPCo) and the gonion plane (GoP), not directly involved in the definition of the 3D neutral track but used for the construction of the latter (Figure 13 and Figure 14).

**Figure 15:**
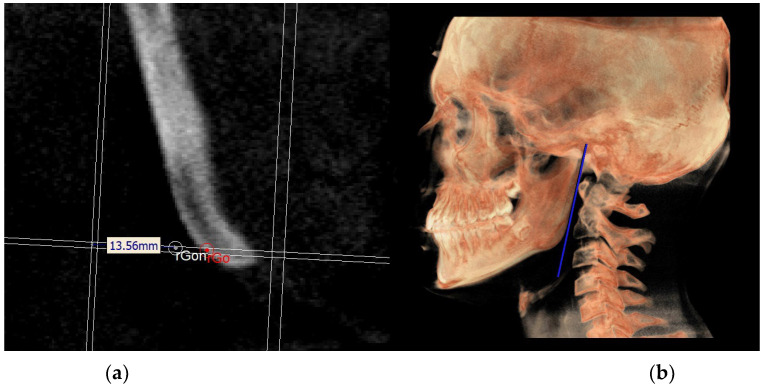
Identification of rGon (**a**) and MRn on volume rendering (**b**).

Once these planes were identified, the distance between the CPCo and the PMn was measured along the GoP and the neutral gonion (Gon) was identified at the middle of this distance. By carrying out this procedure on the right and left side, the rGon and lGon points were located and the MRn plane was identified (Figure 15).

**Figure 16:**
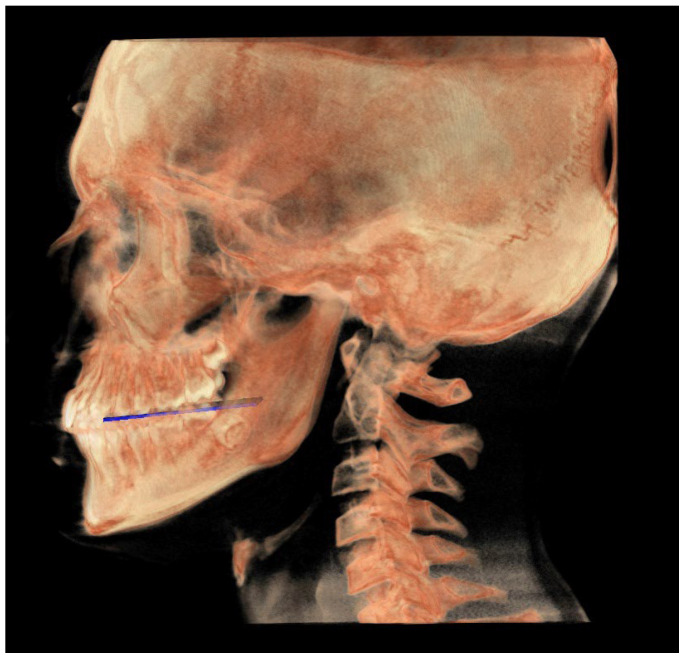
FOPn.

Finally, we proceeded to identify the neutral functional occlusal plane (FOPn) by means of a plane passing through rPoc and lPoc and perpendicular to the PMn (Figure 16).

**Figure 17:**
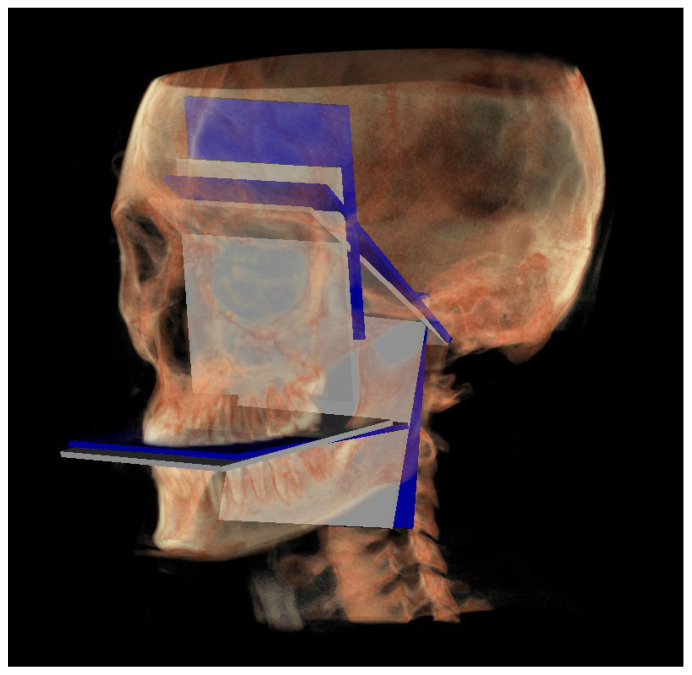
All the planes of the individual track (white) and neutral track (blue).

The following image shows a comparison between the neutral (blue) and individual (white) tracks (Figure 17).

### 2.3. Statistical Analysis

Since the 3D individual track planes (MCF, PM and MR) were identified with different landmarks than the 2D ones, it was necessary to validate them. For this purpose, the average values of the angles between the planes obtained on both 2D and 3D were compared to confirm the null hypothesis of the absence of statistically significant differences between the 2D and 3D individual tracks. The following angles were measured: MCF ^ PM and MCF ^ RM. Since the FOP was identified in 3D with the same points as 2D, it was not necessary to validate it; likewise there was no need to validate the 3D neutral track since this was realized on the CBCT images according to the principles of Enlow’s counterpart analysis [10]. All the 2D angles were automatically measured by the software OrisCeph and all the 3D angles were automatically measured by the software Materialise Mimics with the function “measure and analyze” from the software’s tools menu.

Statistical analysis was performed using the Prism–GraphPad software (Graphpad software, LLC, San Diego, CA, USA). The Kolmogorov–Smirnov normality test was applied for each variable to check whether data were normally distributed. Since data were normally distributed, the paired Student’s *t*-test was performed. The level of statistical significance was *p* < 0.05.

## 3. Results

A total of 18 cephalograms were extracted from the CBCT images and evaluated. Both 2D Enlow’s counterpart analysis and 3D analysis were traced on lateral cephalograms extracted from CBCT and on the CBCT images, respectively.

The average values obtained from the measurements of the following 2D and 3D angles were calculated: MCF ^ PM and MCF ^ MR. Table 6 shows the means and standard deviations of each variables first in 2D and then in 3D.

Once the Kolmogorov–Smirnov test was performed and verified that the sample was normally distributed, the paired Student’s *t*-test was applied.

The gap between the values of each variable was small and no statistically significant difference was highlighted by the paired Student’s *t*-test (*p* > 0.05), confirming the null hypothesis and proving the validity of the proposed new method for the 3D analysis of the neutral track.

## 4. Discussion

The use of CBCT is an important supplement for orthodontic diagnosis and treatment, as it allows the orthodontist to improve diagnosis, especially in situations of complex anatomy and in the presence of asymmetries.

Craniofacial disharmonies are considered a predisposing factor for respiratory disorders. Understanding the rotational factor of the patient’s skeletal pattern is very important, for example, as an open bite accompanied by clockwise mandibular rotation can influence the airway size [13]. Similar craniofacial features were found in patients with pediatric rheumatologic conditions such as juvenile idiopathic arthritis (JIA) [14]. Nowadays, thanks to the use of CBCT, it is possible to analyze skeletal and airway anomalies in a broader way, developing personalized treatment plans for the individual patient. Orthodontics is therefore not limited only to aesthetic but is a medical discipline that can improve the quality of patients’ lives.

Despite the increasingly widespread use of CBCT in daily clinical practice, there is still not much literature on the use of this technology to perform cephalometric diagnoses. The transition from 2D to 3D cephalometry still requires further studies as measurements commonly performed in 2D may differ when transferred to 3D, e.g., a line taken in 2D becomes a 3D plane on CBCT images and so, as defined by Tanna et al. [15], further studies are necessary in order to define new points, new planes and new measurements.

This study was created with the aim of integrating what has already been performed previously by our research group [7] in order to transfer the Enlow analysis into 3D and to be able to provide the current literature with further points, measurements and planes for use in 3D cephalometric diagnosis.

One of the main innovations in this analysis is the method used to transfer the neutral Enlow track in 3D. Although nowadays this is complex to perform manually, since its construction totally depends on the identification of the SEn point, we assume that, once our method has been integrated into the cephalometric analysis software, the operator should simply identify the SEn point and the software analysis tools will be able to independently calculate the neutral 3D landmarks and completely build an autonomous mode within the 3D neutral path. This is possible thanks to the use of artificial intelligence (AI) which in recent years has been increasingly tested in the orthodontics field [16,17]. Several studies affirmed greater accuracy and reduced time and human effort on automated landmark detection when compared to the traditional method [18,19,20,21,22,23], while other studies investigated the use of artificial intelligence (AI) on CBCT for automated 3D cephalometric landmark detection showing that the results are accurate and less time-consuming compared to manual analysis [24,25,26,27]. In recent years, the shift in orthodontic planning is leaning towards soft tissue-drive diagnostics: despite the fact that CBCT is a useful method to evaluate skeletal components, it is of limited value in the assessment of the facial soft tissue, which moreover is deformed by the tools used for the scanning procedure such as forehead restraints and chin rests. To improve the lack of soft tissue data provided by CBCT, 3D facial scans using true depth technology such as smartphones have been integrated in clinical practice. The use of AI is widely spread for the diagnosis of mobile phone 3D facial scans and this combination is a powerful tool for early diagnostics [28].

Having a 3D representation of the comparison between individual and neutral tracks has a double advantage: it allows the clinician to facilitate his evaluations during the diagnostic phase and it improves communication with the patient, who can see a graphic and intuitive representation of its malocclusion, in relation to an ideal model.

This 3D cephalometric analysis could be a useful tool also for maxillofacial surgeons in orthognathic surgery planning and assessing its outcome.

Unlike the traditional method performed on teleradiographs, the landmarks needed for this proposed analysis are only anatomical points, thus increasing the precision of the skeletal evaluation. For example, the Basion point is of difficult localization on cephalometric analysis performed on teleradiographs; instead, it is very simple to identify in the axial view of the scan when performing a cephalometric analysis on CBCT.

Due to the anatomical positions of the points identified in this 3D analysis, a large FOV is necessary and a reduced FOV cannot be used. This analysis could be useful for more complex cases, namely situations wherein teleradiographs could show superimpositions (i.e., skeletal asymmetries) such as in patients who will undergo orthognathic surgery or in cases where CBCT is already prescribed for other clinical reasons (i.e., dental inclusions).

Different DICOM file processing software can be used to perform three-dimensional cephalometric measurements, although in the current literature, there are studies that compare the volumetric measurements of the airways obtained with the different software [29,30,31]. There are no studies that perform these comparisons for the 3D cephalometric measurements. This gap will have to be filled with further studies in the future to obtain precise protocols for 3D cephalometric diagnosis.

A limitation of this study is that the sample investigated is exclusively composed by skeletal Class I subjects, so further studies with a larger sample size and skeletal class II and III subjects need to be performed to confirm the results of this research and allow a more comprehensive understanding of the effectiveness of the proposed method in different populations.

Another limitation of the proposed method is the difficulty in analyzing the occlusal plane in patients with amalgam filling or bridges due to CBCT artifacts that increase errors in landmark identification. This limitation can be overcome by taking a CBCT scan of the patient and combining it with an intraoral scan.

Future investigations could be performed based on this preliminary study to identify all the landmarks of the vertical counterpart analysis proposed by D.H. Enlow and to achieve a complete 3D counterpart analysis.

## 5. Conclusions

To the authors’ knowledge, this is the first study to propose a method as to how to perform the neutral track analysis in 3D and is also the first study proposing a method to analyze the rotational factor of the craniofacial structures on CBCT.

The proposed method could be useful for:

- Orthodontists in the diagnostic phase when there is the need to assess at which level the malocclusion is located;
- Orthognathic surgery planning.

Despite the fact that the absence of statistically significant differences was highlighted, our cephalometric analysis proposal should be verified with further studies involving a bigger sample and different subject populations.

The result of this study represents another step towards a complete 3D Enlow analysis.

## Figures and Tables

**Table 1:** Landmarks and lines identified in the 2D Enlow’s individual track.

Landmarks	
SE	Sphenoethmoidal junction: the intersection of the averaged image of the right and left shadows of the great wings of the sphenoid with the floor of the anterior cranial fossae
Ar	Articulare: the point of intersection between the posterior margin of the ramus and the outer margin of the cranial base
Ptm	Pterygomaxillary fissure: the lowest point in the contour of the pterygomaxillary fissure formed anteriorly by the retromolar tuberosity of the maxilla and posteriorly by the anterior curve of the pterygoid process of the sphenoid bone
Go	Gonion: the geometric construction point given by the intersection of two lines wherein one passes from Me to the lower most point of the mandibular corpus and the other passes from Ar to the posterior most point of mandibular ramus
Poc	Posterior occlusal contact: the most supero-distal contact point of the first molars
Aoc	Anterior occlusal contact: the most mesial contact point of the first premolars or first deciduous molars
**Lines**	
MCF	Middle Cranial Floor, SE—Ar
PM	Pterygo-Mandibular plane, SE—Ptm
MR	Mandibular Ramus, Ar—Go
FOP	Functional Occlusal Plane, Poc—Aoc

**Table 2:** Landmarks and lines identified in the 2D Enlow’s neutral track.

Landmarks	
SEn	SE neutral: the point of a circumference with the center in Ar and the radius equal to the MCF, in which an angle of the skull base equal to 40.3° is obtained
Gon	Gonion neutral: the point located at the same level of the Go and halfway between the PMn line and its parallel passing through Ar
**Lines**	
PMn	PM neutral: the line parallel to the PM forming at the point SEn an ideal angle of 40.3° with the MCFn
MCFn	MCF neutral, Ar—Sen
MRn	MR neutral, Ar—Gon
FOPn	FOP neutral, plane perpendicular to the PMn passing through Poc

**Table 3:** All the points of the 3D individual track.

Point.	X (Left to Right) Sagittal View	Y (Superior to Inferior) Axial View	Z (Posterior to Anterior) Coronal View
Middle cranial floor, right side (rMCF)	Point in superior and endocranial surface where greater wings of sphenoid cross anterior cranial floor at posterolateral bony wall of right orbit	Anterior-most point of middle cranial floor (endocranial surface of greater wings of the sphenoid), right side	Point in endocranial surface where greater wing of sphenoid crosses anterior cranial floor at lateral bony wall of right orbit
Middle cranial floor, left side (lMCF)	Point in superior and endocranial surface where greater wings of sphenoid cross anterior cranial floor at posterolateral bony wall of left orbit	Anterior-most point of middle cranial floor (endocranial surface of greater wings of the sphenoid), left side	Point in endocranial surface where greater wing of sphenoid crosses anterior cranial floor at lateral bony wall of left orbit
Basion (Ba)	Most anterior point of foramen magnum	Most anterior point of foramen magnum	Most anterior point of foramen magnum
Right condylion (rCo)	Most posterior point of mandibular condyle, right side	Most posterior point of mandibular condyle, right side	Most posterior point of mandibular condyle, right side
Left condylion (lCo)	Most posterior point of mandibular condyle, left side	Most posterior point of mandibular condyle, left side	Most posterior point of mandibular condyle, left side
Middle point between right and left Co (mCo)	Middle point between the two condylion	Middle point between the two condylion	Middle point between the two condylion
Right gonion (rGo)	Point at inferior border of mandibular angle at mid-distance between posterior-inferior-most point of ramus and inferior-posterior-most point of mandibular body, right side	Middle-posterior-most point of mandibular angle, right side	Middle-inferior-most point of mandibular angle, right side
Left gonion (lGo)	Point at inferior border of mandibular angle at mid-distance between posterior-inferior-most point of ramus and inferior-posterior-most point of mandibular body, left side	Middle-posterior-most point of mandibular angle, left side	Middle-inferior-most point of mandibular angle, left side
Posterior nasal spine (PNS)	Most posterior point of the hard palate	Most posterior point of the hard palate	Most posterior point of the hard palate
Posterior occlusal contact, right side (rPoc)	Most supero-distal contact point of the first molars, right side	/	/
Posterior occlusal contact, left side (lPoc)	Most supero-distal contact point of the first molars, left side	/	/
Anterior occlusal contact, right side (rAoc)	Most mesial contact point between the first premolars, right side	/	/
Anterior occlusal contact, left side (lAoc)	Most mesial contact point between the first premolars, left side	/	/
Middle point between right and left Aoc (mAoc)	Middle point between the rAoc and lAoc	Middle point between the rAoc and lAoc	Middle point between the rAoc and lAoc

**Table 4:** Points of the 3D neutral track.

Point	X (Left to Right) Sagittal View	Y (Superior to Inferior) Axial View	Z (Posterior to Anterior) Coronal View
SE neutral (SEn)	/	Point of a circumference with center in Ba and radius equal to MCFx, in which an angle of the skull base equal to 40.3° is obtained	/
Right SE neutral (rSEn)	/	Point taken arbitrarily on the right side of the SEn on the same arc of circumference	/
Left SE neutral (lSEn)	/	Point taken arbitrarily on the left side of the SEn on the same arc of circumference	/
Middle point MCF (MCFx)	/	Point of intersection between the plane on which the Ba lies and a line joining the left and right MCF	/
Neutral right gonion (rGon)	Point located at the same level of the Go and halfway between the PMn line and the CPCo plane. Right side	/	/
Neutral left gonion (lGon)	Point located at the same level of the Go and halfway between the PMn line and the CPCo plane. Left side	/	/

**Table 5:** Planes of the neutral track.

Planes	
Plane	Description
MCF neutral (MCFn)	Plane passing through the Ba, rSEn and lSEn which forms an angle of 40.3° with the PMn at the SEn point
PM neutral (PMn)	Plane parallel to the PM such as to form an ideal angle of 40.3° with the MCFn at the SEn point
MR neutral (MRn)	Plane passing through the mCo, rGon and lGon
FOP neutral (FOPn)	Plane normal to the PMn passing through rPoc and lPoc
Construction plane through Ba (CPBa)	Construction plane passing through Ba and parallel to the PM
Construction plane through Co (CPCo)	Construction plane passing through the mCo and parallel to the PMn
GoP	Gonion plane: plane passing through rGo and lGo normal to PMn

**Table 6:** Average values of the 2D and 3D angles.

Index	2D (Mean ± SD)	3D (Mean ± SD)	*p* Value ^a^
MCF ^ PM	43.89° ± 4.25°	43.20° ± 4.08°	0.62
MCF ^ RM	119.40° ± 8.40°	118.8° ± 7.18°	0.83

Legend: SD = standard deviation. ^a^ Paired Student’s *t*-test, level of significance was set at *p* < 0.05.

## Data Availability

The data presented in this study are available on request from the corresponding author.
